# Multisensory Perceptual Biases for Social and Reward Associations

**DOI:** 10.3389/fpsyg.2021.640684

**Published:** 2021-03-11

**Authors:** Moritz Stolte, Charles Spence, Ayla Barutchu

**Affiliations:** ^1^Department of Psychology, University of Vienna, Vienna, Austria; ^2^Department of Experimental Psychology, University of Oxford, Oxford, United Kingdom

**Keywords:** self bias, reward, multisensory, shape matching, order effect

## Abstract

Linking arbitrary shapes (e.g., circles, squares, and triangles) to personal labels (e.g., self, friend, or stranger) or reward values (e.g., £18, £6, or £2) results in immediate processing benefits for those stimuli that happen to be associated with the self or high rewards in perceptual matching tasks. Here we further explored how social and reward associations interact with multisensory stimuli by pairing labels and objects with tones (low, medium, and high tones). We also investigated whether self and reward biases persist for multisensory stimuli with the label removed after an association had been made. Both high reward stimuli and those associated with the self, resulted in faster responses and improved discriminability (i.e., higher *d’*), which persisted for multisensory stimuli even when the labels were removed. However, these self- and reward-biases partly depended on the specific alignment between the physical tones (low, medium, and high) and the conceptual (social or reward) order. Performance for reward associations improved when the endpoints of low or high rewards were paired with low or high tones; meanwhile, for personal associations, there was a benefit when the self was paired with either low or high tones, but there was no effect when the stranger was associated with either endpoint. These results indicate that, unlike reward, social personal associations are not represented along a continuum with two marked endpoints (i.e., self and stranger) but rather with a single reference point (the self vs. other).

## Introduction

To what extent are judgments concerning abstract concepts such as power, morality, or the self based on concrete, akin to perceptual, representations? Early work on this topic grew out of the cognitive linguistic theory of conceptual metaphor put forward by Lakoff and Johnson (1980, 1999). These researchers argued that many metaphors operate by mapping abstract concepts into more concrete concepts, reflecting basic physical properties of the world. For example, words with a positive valence are categorized more rapidly when presented at the top of the screen (as compared to when they are presented at the bottom) and responses to words with negative valence are responded to more quickly when the word happens to be presented at the bottom of the screen (as compared to the top) (Meier and Robinson, 2004). Metaphor congruency effects have been demonstrated across a range of conceptual and perceptual representations in binary classification tasks discriminating between moral and immoral terms (Meier et al., 2007), and power vs. powerlessness (Schubert, 2005). Links in conceptual and perceptual representations have also been observed with both visual stimuli (Dehaene et al., 1993; Fias et al., 2001; Fischer, 2003; Nuerk et al., 2004) and across vision and audition (e.g., Rusconi et al., 2006). Such abstract representations can also extend to self-biases and monetary rewards. As yet, however, it is unknown how perceptual representation influences such conceptual hierarchies of the self and rewards.

Several recent studies have investigated the benefits of self-relevant stimuli in human information processing. For example, Sui et al. (2012) demonstrated that forming associations between arbitrary geometric shapes and people (e.g., square – you, circle – friend, triangle – stranger) results in immediate processing benefits for the self-associated shapes as compared to the others: When judging whether a presented shape-label pair matched the initial association or not, participants responded significantly faster and more accurately to matching self-pairs as compared to matching friend- or stranger-pairs. Given its ability to provide an estimate of the self-bias effect free from confounds of familiarity and memory with self-relevant stimulus material (e.g., one’s face or name), the paradigm has subsequently been used in a variety of studies (e.g., Sui et al., 2012, 2013; Humphreys and Sui, 2015; Desebrock et al., 2018; Kim et al., 2019) and has also been adapted to assess possible contributing factors to self-bias effects such as emotion (Stolte et al., 2017) and reward (Sui et al., 2012, 2015; Sui and Humphreys, 2015; Yankouskaya et al., 2017, 2018). The comparison of social and reward processes is important as reward has been implicated as a possible underlying factor for the observed self-prioritization effect (SPE). For example, Northoff and Hayes (2011) proposed that inherent reward value is associated with the self and guides self-biases in perception. Moreover, self and reward have been found to at least partially overlap in behavioral (e.g., Sui et al., 2012) as well as, in neuroimaging studies (e.g., de Greck et al., 2008; Enzi et al., 2009; Yankouskaya et al., 2017), demonstrating shared activations in reward gambling and personal relevance attribution tasks. Moreover, monetary rewards have a natural order in terms of magnitude, and therefore allow for a comparison of possible effects of ‘ordered’ representation within the social hierarchies. However, the question of how social and reward associations contribute and interact with such biases across different sensory systems, has not been investigated previously.

It is also important to investigate self and reward biases across the senses as multisensory processing can have a large enhancing effect on many forms of cognitive and behavioral processing, including speed, accuracy, and memory, and incidental associative learning in matching tasks (e.g., Seitz et al., 2006; Shams and Seitz, 2008; Mitchel and Weiss, 2011; Fifer et al., 2013; Barutchu et al., 2020). Recent studies have also demonstrated that multisensory processes can be influenced by other social factors, such as joint attention and the precence of an experimenter in the testing room (Wahn et al., 2017; Barutchu and Spence, 2020). However, the effect of multisensory processing on incidental learning of self and reward associations, and self-biases are currently unknown. Although self-bias effects have been observed previously in the visual, auditory, and tactile modalities (Schafer et al., 2016), to the best of our knowledge, self biases to multisensory stimuli (i.e., simultaneously presented audiovisual stimuli associated with social and reward labels) have not been investigated. Moreover, to date no study has investigated whether self and reward biases persist with multisensory stimulation in the absence of labels. This is important because, in real life, those objects that are associated with the self or with rewards are usually not labeled as such. For example, in everyday life, people generally identify objects as belonging to themselves and have to maintain this information in memory for identification when encountering them subsequently. Therefore, there is a limit to how much the current understanding of self and reward biases can be generalized to daily life. To bridge this gap in the literature, we investigate self and reward biases in the absence of explicit visual labels following a brief learning association phase.

To be able to compare unisensory and multisensory social and reward categories, two experiments were designed in which the associations in each category were paired with an auditory stimulus that has its own natural order – the same three low (500 Hz), medium (800 Hz), and high (1100 Hz) pure tones were associated and presented simultaneously with social (myself, friend, and stranger) and reward (£18, £6, and £2) labels. A perceptual matching task (as in Sui et al., 2012), where the participants had to judge whether two stimuli were correctly paired (corresponding to the original association made) or not, was used to obtain performance measures of reaction time (RT), accuracy, and perceptual sensitivity such as d-prime (*d’*). Both the self and the reward experiment used the same three conditions: visual only (shape-label pairings), audiovisual-labels (tone-label pairing), and audiovisual-shapes (shape-tone pairings that were associated with labels in a brief learning phase but tested in the absence of the labels). This third condition allowed us to investigate whether audiovisual associations persist in the absence of explicit social and reward labels.

In the two experiments reported here, personally associated stimuli (Experiment 1: myself, friend, stranger), and reward stimuli (Experiment 2: £18, £6, £2), were linked to tones and/or shapes. Following a close inspection of the data, we noted an incidental finding whereby the natural order of the tones interacted with the abstract hierarchies of the self and rewards. Therefore, we ran further exploratory analyses, whereby the participants were allocated into one of three sub-groups depending on the assignment of the ‘medium tone’ to a specific reward or social label, while the assignments of the low and high endpoints of the tones were still counterbalanced across the other two labels within each group (see section Methods for details). Similarly, the assignment of the labels to geometric shapes was also counterbalanced within each of the sub-groups of participants. Thus, participants were allocated into three groups according to the particular tones the shapes and labels were paired with – with the tones either falling at the end (high and low) or the center (medium) of the scale. In those cases where ‘central’ values along the social or reward stimuli (i.e., friend or £6) aligned with the medium tone value (i.e., 800 Hz), we assumed that there is a midpoint match across the stimuli. In contrast, for other stimulus combinations, the medium tone could align with one end of the personal or reward stimuli (e.g., self/£18 to medium tone, or stranger/£2 to medium tone), so that the personal and reward labels would not align with the physical dimension of the tone. This design enabled us to analyze whether relative tone-order influenced self-bias and reward-bias effects across all participants.

We hypothesized, first of all, that previously observed self- and reward biases (using perceptual matching to geometric shapes) would be replicated for associations with tones and audiovisual stimuli even in the absence of a label. Secondly, we explored the interactions of self and reward with tone order. If hierarchical information is present in the social and reward dimensions, it should interact with the ordered perceptual representations of the tones. Finally, this effect was expected to differ between the social and reward experiments if they rely on separate underlying cognitive mechanisms.

## Experiment 1

### Method

#### Participants

Thirty-six healthy adults (nineteen females, between 20 and 32 years of age, median = 23 years) with normal or corrected to normal vision took part. All gave written informed consent prior to the experiment in accordance with the local ethics committee. The participants were paid for their time. Sample size was based on a previous study using a similar shape-matching paradigm (Stolte et al., 2017) and a power calculation to obtain a medium effect size (*f* = 0.25), at a minimum power of 0.80 (α = 0.05).

#### Stimuli

The visual stimuli consisted of three labels (*Myself*, *Friend*, and *Stranger*) and three geometric shapes (circle, square, and hexagon; 3.8° × 3.8° visual angle). The labels were presented in white (3.5° below the fixation point) and the shapes in light gray on a mid-gray background at fixation). The auditory stimuli were three pure tones of low, medium, or high frequency (500, 800, and 1100 Hz, respectively, 75 dB), played through a central loudspeaker placed below the screen. The stimuli were created in Matlab (R2010a) using the Psychophysics Toolbox (Brainard, 1997; Pelli, 1997) and presented on a 21-in. monitor (1920 × 1080 resolution, at 100 Hz).

#### Procedure

Before the main experiment, in a pre-learning phase, each label was presented together with one of the three tones, and one of the three shapes. Participants had to remember the shape-tone-label stimuli by thinking of each tone and shape as representing the specific person assigned to it by the label (i.e., myself, friend, or stranger). In practice, each shape-label pair was presented once for 5 s while the assigned tone was simultaneously repeated (at 100 ms intervals) for the same total duration. The order of presentation in the learning phase and the particular pairing of a tone and shape to self, friend, or stranger was counterbalanced across participants; thus, the participants could be separated into three sub-groups depending on the pairing of the medium tone (MT) to a specific label (i.e., MT Self = *Myself* associated with medium tone; MT Friend = *Friend* associated with medium tone; MT Stranger = *Stranger* associated with medium tone), while the assignment of the low and high tones to the remaining labels was counterbalanced across participants within each group.

The main experiment was conducted following the initial associative learning stage. Each trial began with a white fixation point presented at the center of the screen for 650 ms. After a variable delay of 100–400 ms, pairs of stimuli were presented simultaneously for 100 ms: either a shape together with a label, a tone with a label, or a shape with a tone. Following stimulus presentation, the screen remained blank until a response was made (maximum 3 s). Immediately after each response, feedback was provided on the screen (for 400 ms), indicating a correct or incorrect response or giving a warning (“*Too Slow!”*) for reaction times (RTs) that were greater than 1000 ms. This design encouraged participants to respond as rapidly as possible while still allowing for responses slower than 1000 ms to be collected. On each trial, the stimulus pair (shape-label, label-tone, or shape-tone) could either conform to the original combination from the associative learning stage, or it could be a recombination of two non-matching stimuli. Within each block, matching and non-matching trials were presented randomly with equal probability (i.e., randomly interleaved). On each trial, the participants were instructed to judge whether the two stimuli matched or not by pressing one of two responses buttons as rapidly and accurately as possible. Each participant completed eight blocks of 72 trials; thus performing 32 trials in each of the 18 conditions (shape-label, tone-label, and shape-tone matching and mismatching trials for self, friend and stranger associations).

#### Analyses

Motor RTs and accuracy measures were recoded. Accuracy measures were used to calculate *d’* scores by counting correct responses in matching trials as hits and incorrect responses in non-matching trials as false alarms. The pattern of results for accuracy and *d’* measures were very similar, therefore, here we only present *d’* analyses.

Significantly lower self-biases were noted in the tone conditions suggesting that the order of the tones interacted with the self-associations. Therefore, for each experiment we grouped participants according to the label allocated to the middle tone. We analyzed the data using a 3(association: self, friend, and stranger) × 3(stimulus type: Shape + Label, Tone + Label, and Shape + Tone) × 3(group: MT self = medium tone with self association, MT Friend = medium tone with friend association; MT Stranger = medium tone with stranger association) mixed ANOVA. Significant interaction effects were followed-up with *post hoc* simple effects analyses. For all analyses, all *p*-values for *post hoc* comparisons were adjusted using Bonferroni correction.

### Results and Discussion

**FIGURE 1:**
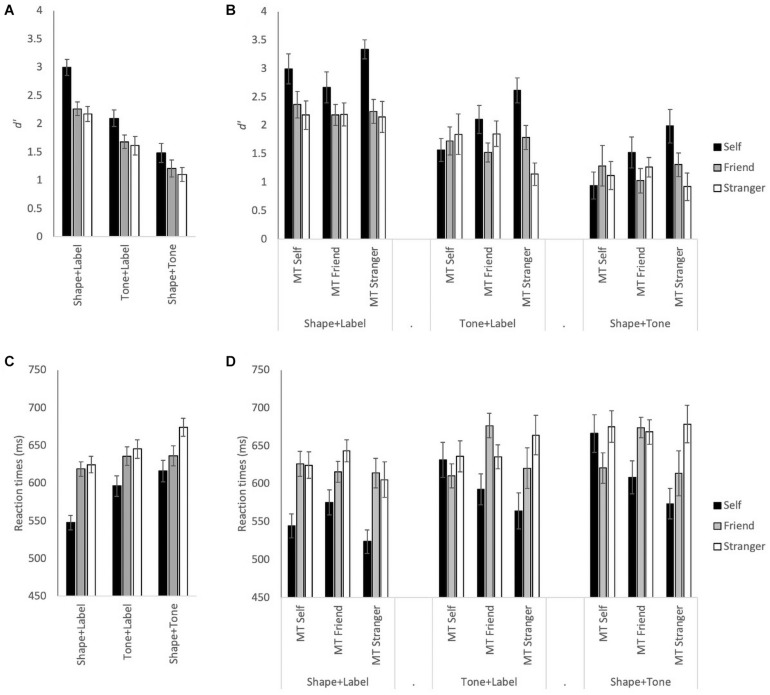
**(A)** Mean d’ for Self, Friend, and Stranger associations for each stimulus type: Shape + Label, Tone + Label, and Shape + Tone conditions, across all participants. **(B)** Mean *d’* with the association and stimulus type conditions split across the three tone groups: medium tone (MT) self, medium tone with self association; MT Friend, medium tone with friend association; MT Stranger, medium tone with stranger association. **(C)** Mean RTs across all participants. **(D)** Mean RT split across the three tone groups. Error bars represent ± 1 *SEM*.

Visual inspection of Figure 1 shows self-bias was observed in both *d’* and RT measures. However, the overall bias was much greater in the visual only condition than in the audiovisual conditions (Figures 1A,C). This was partly due to the hierarchy of the self-associations interacting with the order of tones as shown by allocating participants into groups based on tone and self-associations (see Figures 1B,D). Consistent with our expectations, the results of the Shape + Label condition were not affected by the (tone-)group allocation as there is no natural order to the shapes used. Therefore, as expected, the patterns of *d’* (Figures 1A,B) and RT results (Figures 1C,D) in the visual only Shape + Label condition are very similar before and after group allocation. However, the pattern of results changes significantly in the multisensory Tone + Label and Shape + Tone conditions. Here, the normally observed trend of increased performance for self-associated stimuli (self > friend > stranger) was only preserved when the medium tone was paired with the stranger label or with stranger-associated shape.

See Table 1 for all ANOVA F-statistics for *d’* and RTs; for both the main effects of stimulus type and association were significant (see Figures 1A,C). Overall, significantly higher *d’* measures and faster RTs were observed for self-associations than friend and stranger associations (*p* < 0.001 for all); *d’* measures for the friend and stranger associations did not differ from each other, though RTs were significantly faster for friend than stranger (*p* = 0.002). In addition, significantly higher *d’* and faster RTs were observed for the visual only Shape + Label condition than the multisensory Tone + Label, both of which were faster with higher *d’* measures than the Shape + Tone conditions (*p* < 0.004 for all main effect comparisons).

**TABLE 1:** F-statistics for *d*’ and RTs for Experiments 1 (Exp 1) and 2 (Exp 2) assessed using 3(Association: Self, Friend, and Stranger for Exp 1 or £18, £6, and £2 for Exp 2) × 3(Stimulus Type: Shape + Label, Tone + Label and Shape + Tone) × 3(Group: MT Self or £18 = medium tone with self or £18 association, MT Friend or £6 = medium tone with friend association or £6; MT Stranger or £2 = medium tone with stranger association or £2) mixed ANOVAs for each experiment.

		*d*’	RT
Exp 1 – Self	Assoc	*F(2,66) = 19.21, *p* < 0.001, η*^2^* = 0.37	*F(2,66) = 37.78, *p* < 0.001, η*^2^* = 0.53
	Stim	*F(2,66) = 90.27, *p* < 0.001, η*^2^* = 0.73	*F(2,66) = 29.76, *p* < 0.001, η*^2^* = 0.47
	Group	*F*(2,33) = 0.22, *p* > 0.05, η*^2^* = 0.01	*F*(2,33) = 0.65, *p* > 0.05, η*^2^* = 0.04
	Assoc × ST	*F(4,132) = 3.65, *p* = 0.007, η*^2^* = 0.10	*F(4,132) = 6.02, *p* < 0.001, η*^2^* = 0.15
	Assoc × Group	*F(4,132) = 7.03, *p* < 0.001, η*^2^* = 0.30	*F(4,132) = 5.52, *p* = 0.001, η*^2^* = 0.25
	ST × Group	*F*(4,132) = 0.74, *p* > 0.05, η*^2^* = 0.04	*F*(2,132) = 0.59, *p* > 0.05, η*^2^* = 0.04
	Assoc × ST × Group	*F(8,132) = 2.98, *p* = 0.004, η*^2^* = 0.15	*F(8,132) = 6.69, *p* < 0.001, η*^2^* = 0.29
Exp 2 – Reward	Assoc	*F(2,66) = 3.45, *p* = 0.04, η*^2^* = 0.10	*F(2,66) = 14.34, *p* < 0.001, η*^2^* = 0.30
	Stim	*F(2,66) = 37.65, *p* < 0.001, η*^2^* = 0.53	*F(2,66) = 15.98, *p* < 0.001, η*^2^* = 0.33
	Group	*F(2,33) = 1.98, *p* > 0.05, η*^2^* = 0.11	*F*(2,33) = 1.99, *p* > 0.05, η*^2^* = 0.11
	Assoc × Stim	*F*(4,132) = 0.17, *p* > 0.05, η*^2^* = 0.01	*F(4,132) = 3.48, *p* = 0.01, η*^2^* = 0.10
	Assoc × Group	*F*(4,132) = 2.50, *p* = 0.05, η*^2^* = 0.13	*F(4,132) = 5.48, *p* = 0.001, η*^2^* = 0.30
	Stim × Group	*F*(4,132) = 1.03, *p* > 0.05, η*^2^* = 0.06	*F*(4,132) = 1.19, *p* > 0.05, η*^2^* = 0.07
	Assoc × Stim × Group	*F(8,132) = 4.11, *p* = 0.001, η*^2^* = 0.20	*F(8,132) = 8.91, *p* < 0.001, η*^2^* = 0.35
Exp 1 vs. Exp 2	Order	*F*(1,66) = 0.43, *p* > 0.05, η*^2^* = 0.01	*F*(1,66) = 0.04, *p* > 0.05, η*^2^* = 0.001
	ST	*F(2,132) = 4.74, *p* = 0.01, η*^2^* = 0.07	*F(2,132) = 10.18, *p* < 0.001, η*^2^* = 0.13
	Group	*F*(2,66) = 1.11, *p* > 0.05, η*^2^* = 0.03	*F*(2,66) = 2.23, *p* > 0.05, η*^2^* = 0.06
	Exp	*F(1,132) = 7.83, *p* = 0.007, η*^2^* = 0.11	*F(1,132) = 5.05, *p* = 0.03, η*^2^* = 0.07
	Order × Stim	*F*(2,132) = 0.10, *p* > 0.05, η*^2^* = 0.002	*F(2,132) = 8.27, *p* < 0.001, η*^2^* = 0.11
	Order × Group	*F*(2,66) = 0.44, *p* > 0.05, η*^2^* = 0.01	*F(2,66) = 5.57, *p* = 0.006, η*^2^* = 0.14
	Order × Exp	*F(1,66) = 4.70, *p* = 0.03, η*^2^* = 0.07	*F(1,66) = 14.55, *p* < 0.001, η*^2^* = 0.18
	Stim × Group	*F(4,132) = 3.26, *p* = 0.01, η*^2^* = 0.09	*F(4,132) = 3.89, *p* = 0.005, η*^2^* = 0.11
	Stim × Exp	*F(2,132) = 3.42, *p* = 0.04, η*^2^* = 0.05	*F(2,132) = 1.01, *p* > 0.05, η*^2^* = 0.02
	EXP × Group	*F(2,132) = 11.29, *p* < 0.001, η*^2^* = 0.26	*F(2,132) = 11.25, *p* < 0.001, η*^2^* = 0.25
	Order × Group × Exp	*F(2,66) = 4.25, *p* = 0.02, η*^2^* = 0.11	*F*(2,66) = 2.16, *p* > 0.05, η*^2^* = 0.06
	Order × Stim × Group	*F*(4,132) = 2.42, *p* = 0.05, η*^2^* = 0.07	*F(4,132) = 9.26, *p* < 0.001, η*^2^* = 0.22
	Order × Stim × Exp	*F*(2,132) = 0.04, *p* > 0.05, η*^2^* = 0.001	*F*(2,132) = 0.15, *p* > 0.05, η*^2^* = 0.02
	ST × Group × Exp	*F(4,132) = 5.87, *p* < 0.001, η*^2^* = 0.15	*F(4,132) = 15.53, *p* < 0.001, η*^2^* = 0.32
	Order × Stim × Exp × Group	*F(4,132) = 2.93, *p* = 0.02, η*^2^* = 0.08	*F(4,132) = 3.20, *p* < 0.05, η*^2^* = 0.09

Table also includes F-statistics for mixed four way ANOVAs for both d’ and RTs comparing the two experiments: 3(Order of association: Friend/£6 and Stranger/£2 difference from Self/£18) × 3(Stimulus Type: Shape + Label, Tone + Label and Shape + Tone) × 3(Group: MT Self or £18 = medium tone with self or £18 association, MT Friend or £6 = medium tone with friend association or £6; MT Stranger or £2 = medium tone with stranger association or £2) × 2(Experiment: Self and Reward). Assoc, association; Sim, stimulus type; Exp, experiment; Order, order of association. *Significant F-statistics with p < 0.05.

Importantly, for *d’* and RTs, the three-way interaction between stimulus, association, and group was also significant (see Table 1 and Figure 1B). In the MT self group, self-bias for *d’* was observed for the Shape + Label condition, though Friend and Stranger associations did not differ from each other (see Table 2). For the MT Friend group, only *d’* measures for the self and friend associations significantly differed in the Tone + Label condition (*p* = 0.02). Self-biases were most prominent and *d’* was significantly higher in the MT Stranger group across all types of stimuli (*p* < 0.03 for all); only the friend vs. stranger comparisons failed to reach significance in the Shape + Label and Shape + Tone conditions (*p* > 0.05).

**TABLE 2:** *P*-values for *post hoc* pairwise comparisons using simple effects analyses for significant three-way interactions comparing the association types [S/£18 = self or £18, F/£6 = Friend or £6, Str/£2 = Stranger or £18 for Experiment (Exp) 1 and 2, respectively] and stimulus pairs (Shape + Label = SL, Tone + Label = TL, and Shape + Tone = ST) across middle tone (MT) groups (Group 1 = MT self or MT £18, Group 2 = MT Friend or MT £6, and Group 3 = MT Stranger or MT £2, for Experiments 1 and 2, respectively).

				Association pairwise comparisons							Stimulus pairwise comparisons			
				Self Exp 1		Reward Exp 2					Self Exp 1		Reward Exp 2	
Group	Stim	Assoc pairs		*d*’	RTs	*d*’	RTs	Assoc	Stim pairs		*d*’	RTs	*d*’	RTs
1	SL	S/£18	F/£6	0.003*	<0.001*	0.071	0.042	S/£18	SL	TL	<0.001*	<0.001*	0.001*	<0.001*
			Str/£2	0.001*	<0.001*	0.952	0.977			ST	<0.001*	<0.001*	<0.001*	<0.001*
		F/£6	Str/£2	0.366	0.894	0.062	0.015*		TL	ST	0.001*	0.009*	0.013*	0.771
	TL	S/£18	F/£6	0.446	0.186	0.178	0.029	F/£6	SL	TL	0.003*	0.312	0.406	0.325
			Str/£2	0.297	0.785	0.005*	<0.001*			ST	<0.001*	0.741	0.003*	0.433
		F/£6	Str/£2	0.643	0.117	0.034	0.003*		TL	ST	0.027	0.484	0.014*	0.92
	ST	S/£18	F/£6	0.155	0.018	0.309	0.054	Str/£2	SL	TL	0.16	0.423	0.44	0.777
			Str/£2	0.429	0.679	0.184	0.001*			ST	<0.001*	0.003*	<0.001*	0.107
		F/£6	Str/£2	0.374	0.005*	0.587	0.056		TL	ST	0.003*	0.017	<0.001*	0.076
2	SL	S/£18	F/£6	0.02	0.002*	0.73	0.493	S/£18	SL	TL	0.012*	0.248	0.76	0.208
			Str/£2	0.04	<0.001*	0.747	0.009*			ST	<0.001*	0.061	0.002*	0.256
		F/£6	Str/£2	0.972	0.048	0.999	0.001*		TL	ST	0.001*	0.22	<0.001*	0.002*
	TL	S/£18	F/£6	0.008*	<0.001*	0.029	<0.001*	F/£6	SL	TL	0.002*	<0.001*	0.083	0.038
			Str/£2	0.331	0.022*	0.63	0.441			ST	<0.001*	0.001*	0.001*	0.089
		F/£6	Str/£2	0.194	0.015*	0.021	<0.001*		TL	ST	0.013*	0.86	0.032	0.868
	ST	S/£18	F/£6	0.046	0.001*	0.626	0.149	Str/£2	SL	TL	0.158	0.586	0.574	0.556
			Str/£2	0.247	0.01*	0.048	0.047			ST	<0.001*	0.118	0.037	0.067
		F/£6	Str/£2	0.221	0.746	0.009*	<0.001*		TL	ST	0.013*	0.043	0.018	0.084
3	SL	S/£18	F/£6	<0.001*	<0.001*	0.3	0.216	S/£18	SL	TL	0.002*	0.009*	0.753	0.596
			Str/£2	<0.001*	<0.001*	0.675	0.028			ST	<0.001*	0.006*	0.056	0.326
		F/£6	Str/£2	0.637	0.514	0.141	<0.001*		TL	ST	<0.001*	0.47	0.06	0.043
	TL	S£18	F/£6	<0.001*	0.001*	0.071	0.001*	F/£6	SL	TL	0.027	0.68	0.461	0.14
			Str/£2	<0.001*	<0.001*	0.031	0.002*			ST	0.001*	0.983	0.008*	0.813
		F/£6	Str/£2	0.013*	0.011*	0.355	0.726		TL	ST	0.016	0.647	0.029	0.155
	ST	S/£18	F/£6	0.007*	0.035	0.008*	0.519	Str/£2	SL	TL	<0.001*	<0.001*	0.001*	<0.001*
			Str/£2	<0.001*	<0.001*	0.01*	0.063			ST	<0.001*	<0.001*	<0.001*	<0.001*
		F/£6	Str/£2	0.05	0.001*	0.701	0.111		TL	ST	0.332	0.359	0.087	0.855

Asterisks (*) = significant p-values following a Bonferroni correction.

A similar pattern of results was observed for RTs (see Table 1 and Figure 1D). For the MT self group, significant RT gains were observed for self, compared to both friend and stranger, in the visual only Shape + Label condition (*p* < 0.01), however, RTs for self associations did not differ significantly from friend and stranger in the Tone + Label and Shape + Tone conditions. In the MT Friend group, significantly faster RTs were observed for the Shape + Label condition only for friend associations (*p* < 0.01). In the MT Friend and MT Stranger groups, significantly faster RTs were observed for the Shape + Label condition for the self and stranger associations, but not for the friend association (see Table 2).

In summary, analysis of the results of Experiment 1 demonstrated self-bias effects under both visual only and multisensory conditions. Nevertheless, the effect of self-bias was higher in the visual only than the multisensory conditions, suggesting that it may be easier to form visual associations with the self. Although the self-bias effect was smaller, it persisted even in the absence of association labels (Shape + Tone condition). The results of Experiment 1 also indicate clear differences for the self-bias effect depending on the specific tones that were assigned to each label: When stranger was associated with the middle tone, performance gradually decreased from self to friend to stranger, reflecting the overall pattern of results for the self-bias experiment and the pattern usually observed when shapes are matched to social labels (consistent with e.g., Sui et al., 2012). There was also a self-advantage relative to friend when the self was assigned a tone at the end of the tone dimension (i.e., the low or the high tone) and friend was assigned to the medium tone, although, in this case there was also a relative benefit for assigning stranger to the other end of the tone dimension, so that performance on stranger fell between that for the self and friend. When the self was associated with the medium tone, the self-bias effect was eliminated, and there was also no advantage for the stimuli that were assigned to the ends of the tone dimension (friend and stranger).

## Experiment 2

The second experiment investigated the effects of associating a specific reward (£18, £6, or £2), instead of personal labels, to the same shapes and tones used in Experiment 1 (see Sui et al., 2012). In order to be able to compare the two experiments, all other parameters remained the same (with the exception of explicit feedback on the amount of rewards earned after each block).

### Method

#### Participants

Thirty-six new, healthy adults (26 females, 19–33 years of age, median = 22.5 years) with normal or corrected to normal vision participated for payment. All gave written informed consent prior to the experiment in accordance with the local ethics committee.

#### Stimuli and Procedure

The stimuli and procedure were identical to those used in Experiment 1 except that the labels used were now £18, £6, and £2 (instead of *Myself*, *Friend*, and *Stranger*). In addition, the participants were instructed that they would earn a small percentage of the displayed reward value for every trial they answered correctly. There was no reward given on those trials without a reward label (i.e., on the trials with shape-tone pairs). The participants were instructed to try and maximize the rewards earned and were given an explicit example explaining the reward structure: They were instructed that for a correct trial with the £18 label they would receive nine times as much as for a correct trial with the £2 label. After each block of trials, the total reward earned for that block was displayed on screen. On average, the participants earned £0.84 per block (*SD* = 0.11). As in the previous experiment, the assignment of tones to rewards and shapes was counterbalanced across participants, resulting in three sub-groups that shared the same medium tone to reward value assignment (while assignment of low and high tones to the remaining reward values was counterbalanced within each group).

### Results and Discussion

**FIGURE 2:**
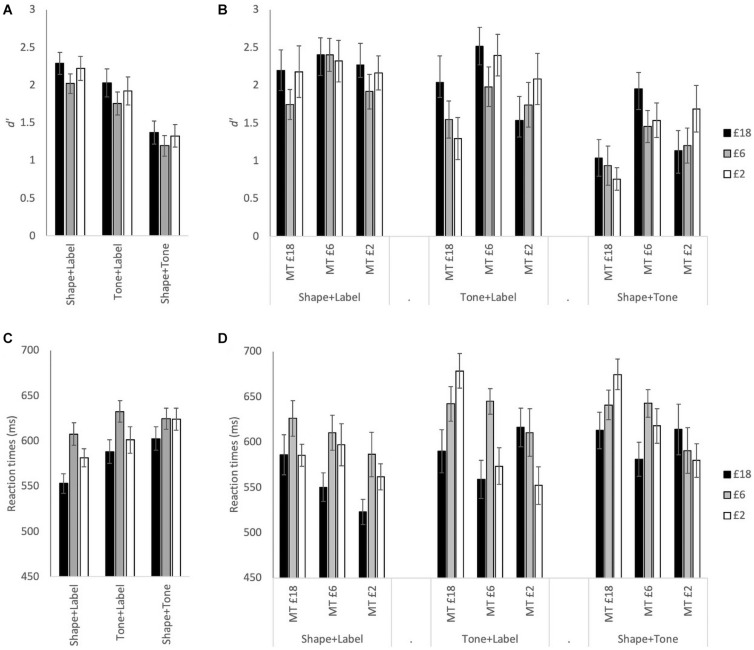
**(A)** Mean *d*’ for £18, £6, and £2 reward associations for each stimulus type: Shape + Label, Tone + Label, and Shape + Tone, across all participants. **(B)** Mean *d’* with the association and stimulus type conditions split across the three tone groups: MT £18 = medium tone with £18 association, MT £6 = medium tone with £6 association; MT £2 = medium tone with £2 association. **(C)** Mean RT across all participants. **(D)** Mean RT split across the three tone groups. Error bars represent ± 1 *SEM*.

Figure 2 illustrates RT and *d’* measures for reward associations which were analyzed with a 3(association: £18, £6, and £2) × 3(stimulus type: Shape + Label, Tone + Label, and Shape + Tone) × 3(group: MT £18 = medium tone with £18 association, MT £6 = medium tone with £6 association; MT £2 = medium tone with £2 association) mixed ANOVA (see Table 1 for F-statistic results). For both *d’* and RTs, the main effects of association and stimulus type were significant (see Table 1). For the £6 association, RTs were significantly slower and *d’* were lower than both £2 and £18 (see Figure 2A). RTs were significantly faster and *d’* was higher for the visual only Shape + Label stimuli than for both of the multisensory stimuli; performance for both RTs and *d’* was significantly worse in the absence of labels (Shape + Tone).

The three-way interactions were also significant (see Table 1). The most prominent changes in *d’* across the different stimulus types were observed in the MT £18 group with *d’* significantly higher in the visual Shape + Label condition than the multisensory stimulus types (i.e., Tone + Label and Shape + Tone) (Table 2); Shape + Label and Shape + Tone conditions did not differ significantly from each other for the £6 and £2 associations. In the MT £6 group, *d’* was higher for stimuli associated with £18 compared to £2 but only for the audiovisual Shape + Tone condition. Shape + Label and Shape + Tone conditions for the £6 and £2 associations also differed from each other. In contrast, in the MT £2 group, the different stimulus types did not significantly differ from each other across any of the reward associations (see Table 2).

Reaction times were also affected by reward association and group allocation (see Table 1 and Figures 2C,D). In the MT £18 group, RTs for the £18 association were significantly faster than the £2 association in the multisensory conditions, while in the visual only Shape + Label condition the £18 association was significantly faster than the £6 association. In the MT £6 group, RTs were significantly faster for the £18 association than the £6 association for all stimulus types. In the MT £2 group, RTs were significantly faster for the £18 association than the £6 association in the visual Shape + Label and the multisensory Tone + Label conditions (see Table 2).

In summary, similar to self-bias, reward-bias was affected by pairing the reward-associated stimulus (label or shape) with tones that differed in their relative frequency, although the pattern of effects differed. In the Label + Shape condition, there was an advantage for the high and low rewards relative to the medium reward condition. In itself, this result suggests that reward-associations are relatively strongly affected by the end of the reward dimension – there was an advantage conveyed by being at the end of the reward dimension; for the low reward association as well as for the high reward association. This proposal is supported by the strong modulatory effect of pairing the reward association with the tone. Specifically, when the medium reward was assigned to the medium tone, there was an advantage for both the high reward and the low reward conditions, which did not differ. When the low reward was assigned to the medium tone, there was an advantage for high relative to medium rewards and for medium relative to low rewards. When the high reward was assigned to the medium tone, there was a reversal of the high reward advantage, so that low reward advantages now were facilitated relative to the high reward condition; The medium reward condition (assigned to the medium position along the reward dimension) was not advantaged relative to the high reward (tied to the medium position along the tone dimension).

#### Self vs. Reward Bias

Experiments 1 and 2 showed different patterns of self versus reward biases across the stimulus types, particularly for the multisensory stimuli; the physical hierarchical organization of the tones interacted differently with the hierarchy in the self and reward dimensions. Therefore, we ran the following additional analysis in order to directly compare the results from the two experiments and investigate whether self and reward biases differed significantly from each other. We calculated ‘gain’ measures for self (Experiment 1) and high rewards (Experiment 2) as an indicator of biases in *d’* and RT by subtracting performance measures related to self and the high reward value of £18 from the ‘mid’ value categories of friend and £6, and the ‘low’ value categories of stranger and £2. Thus, measures related to conditions at the high end of each hierarchy (i.e., self and £18) were subtracted from the middle and low ends in order to compare the magnitude of observed self- and reward-biases. Note that calculations were adjusted so that positive values reflect gains (improvements in discriminability or RT speed) in the self and the high reward conditions, while negative values reflect costs or decreases in performance (lower *d’* or slower RTs).

**FIGURE 3:**
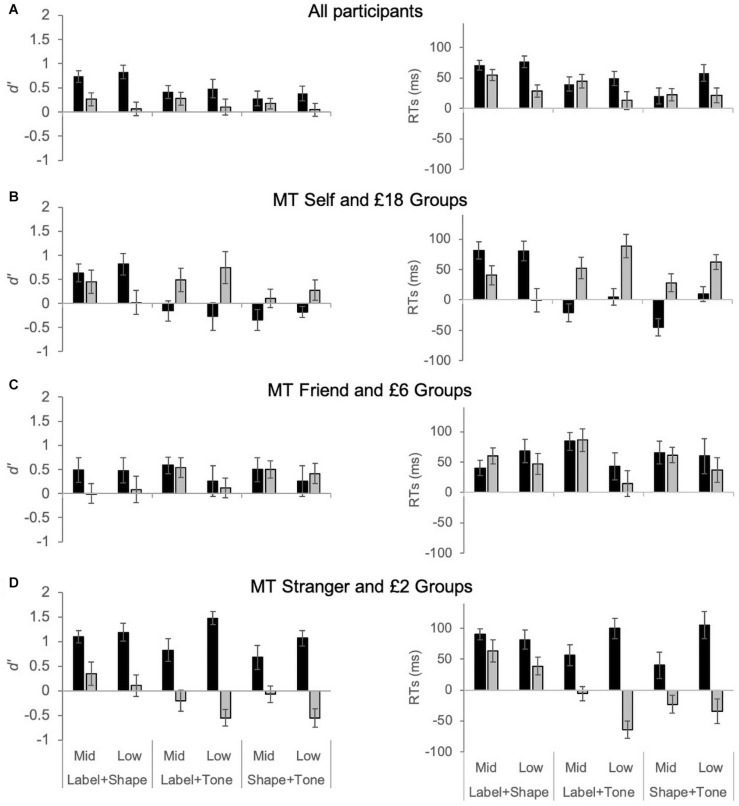
Gain measures for *d*’ and RT (ms) in Experiment 1 for self bias (black bars) and Experiment 2 for reward bias (gray bars). For the self bias experiment ‘mid’ and ‘low’ were defined as the Friend and Stranger association conditions, respectively. For the reward bias experiment, ‘mid’ and ‘low’ were defined as the £6 and £2 association conditions, respectively. Data presented include all participants **(A)**, or are divided into middle tone (MT) groups: MT Self and MT £18 **(B)**, MT Friend and MT £6 **(C)**, and MT Stranger and MT £2 **(D)**.

As illustrated in Figure 3, when all of the participants are considered together, gains are observed for self and high reward across all conditions (Figure 3A). However, the pattern of results changes significantly for the multisensory stimuli when ‘gain’ measures are considered separately across the three tone groups: MT self and £18, MT friend and £6, and MT Stranger and £2 (Figures 3B–D). As can be seen in Figure 3C, self and reward biases for visual and multisensory stimuli can be very similar in magnitude if the ‘middle tone’ is aligned with the midpoints of the conceptual hierarchies of self (i.e., friend) and reward (i.e., £6). However, if the middle tone is aligned with either extreme end of the self or reward category, than the pattern of results changes significantly, as shown below (see also Figures 3B,D).

Here, we present the analysis of the self and reward gains in *d’* and RTs using a 2 (Experiment: self vs. reward) × 2(order of association: mid vs. low comparison to high) × 3(stimulus type: Shape-Label, Label-Tone, Shape-Tone) × 3(group: MT self/£18 = medium tone with self/£18 association, MT Friend/£6 = medium tone with Friend/£6 association; MT Stranger/£2 = medium tone with Stranger/£2 association) mixed ANOVA. The ANOVA was followed-up with simple pairwise planned contrasts, with Bonferroni corrections, to compare self and reward biases across the group, association, and stimulus type conditions.

For both *d’* and RTs, the mixed ANOVAs showed significant four-way interaction effects (see Table 1 for all F-statistic results). Planned contrasts comparing gain measures for *d’* and RTs showed no significant differences between the self and reward experiments for the MT Friend or £6 group – i.e., when the middle tone was paired with the Friend and £6 values (see Figure 3C). For the MT Self/£18 groups, gain measures were significantly higher in the self than reward experiment for both multisensory stimuli (*p* < 0.01 for all pairwise comparisons). The opposite effect was observed for the MT Stranger/£2 groups; for the multisensory stimuli gain measures were significantly higher in the reward than the self experiment for the Tone + Labels condition (*p* < 0.04 for all).

In summary, as expected, group allocation based on tone-order had a minimal effect on gain measures for the visual only stimuli (as the geometric shapes no natural hierarchical order). The self and reward gain measures for the multisensory stimuli, however, were affected by the natural order of the tones. When the middle tone was allocated to the mid values of the self and reward hierarchy (i.e., Friend and £6 association), *d’* and RT gains were similar in the reward and self experiment. However, allocating the middle tone to the high end of the dimensions (Self and £18) resulted in gain in the reward experiment and costs or no gains in the self experiment for the multisensory stimuli. The opposite effect was observed when the low ends (Stranger and £2) were associated with the middle tone, with self associations leading to gains, and the high £18 reward associations resulting in costs. This result provides strong evidence that self and reward biases interact significantly differently with naturally existing physical orders like tone frequency.

## General Discussion

Overall, pooling the data from Experiment 1 across all shape-label-tone pairings revealed an advantage for self over friend and friend over stranger associations; this matches previous results for the perceptual matching of shape-label pairs (Sui et al., 2012). However, this study is the first to demonstrate that such self-biases can be multisensory in nature, and persist in the absence of labels once the association has been formed between multisensory stimuli (e.g., for shape-tone pairs).

In contrast, for reward, the overall results revealed a cost for the medium (£6) as compared to high (£18) and low rewards (£2) showing a very different order effect compared to the hierarchical structure of the social dimension. This result in itself suggests that reward and self associations are influenced differently. Here, for the first time, we also present an incidental finding of how ordinal information within social and monetary reward can interact with the relative order of the physical frequency of tones. We used a simple perceptual matching task where elements in both the self and rewards were paired to the same three tones that differed only in terms of their frequency, showing a complex interplay between these representations. There can be an advantage for stimuli positioned not only at the positive end of that dimension (high reward) but also for stimuli positioned at the negative end of the dimension (low reward). These results suggest that self- and reward-biases may be guided by different mechanisms: unlike for rewards, there appears to be a stronger ‘natural’ ordering of self over friend over stranger for personal associations.

Performance for both, personal associations and reward associations was affected by linking these associations with a stimulus varying along a physical dimension – tone frequency order in this case. For both types of association, there were costs due to assigning stimuli to the medium position along the tone dimension (i.e., the medium tone). Furthermore, depending on the assignment of a specific personal label or reward value to the medium tone, unique patterns of performance emerged which were qualitatively very similar in the two experiments (except for one of the three assignments): (i) Assigning the stranger or the low reward to the medium tone led to a clearly ordered set of data whereby the self and high reward was advantaged relative to the friend and medium reward, which was advantaged relative to the stranger and low reward condition, respectively; (ii) Assigning the friend or medium reward to the medium tone led to advantages for self and high reward and for stranger and low reward as compared with the friend and medium conditions, respectively; (iii) Assigning the self to the medium tone eliminated the differences between the personal label conditions altogether. For the reward conditions, assignment of the high reward to the medium tone resulted in facilitation for the low as compared to the high reward condition.

The contrast between the personal association result and the reward association result, when the middle tone was assigned to the self and the high reward, suggests that assigning the self to the end of the personal association dimension had a stronger impact than the assignment of high reward to the end of the reward dimension. In the case of personal association, the effect of self as an endpoint of the dimension appears to equate to the endpoints in the tone dimension. For the reward association, however, the effect of the highest reward as the endpoint of the dimension was overridden by the tone effect. Indeed this could be due to the nature of the overall hierarchy of the stimuli across the three dimensions (e.g., conceptually self, reward, or the highest tone always positioned on top of a continuum), or it could be due to a difference in how the stimuli are conceptualized relative to each other (i.e., conceptualized as more than or less than relative to each other – the middle tone is higher than the low tone but lower than the high tone) (Stevens, 1971). Further research is needed in order to distinguish between these possibilities.

The results fit with the association tasks being affected by ‘natural’ reference dimensions, which could either align or misalign with the physical dimension of tone frequency to modulate performance. For the reward association task, this is the perceptual hierarchy of magnitude. Given that, in the Shape-Label task, the high and low reward conditions were facilitated relative to the medium reward condition, the ends of the magnitude dimension appear to be weighted relatively equally. This also allows the low reward condition to benefit relative to the high reward condition, when the high reward condition was linked to the medium tone. For personal relations, the reference dimension is more conceptual in nature, but here there appears to be a stronger weighting for the positive end of that dimension (the self) than the negative end (the stranger), since (i) in the shape-label condition there was an ordering of performance from the self (most advantaged) to the friend to the stranger, and (ii) there was not a reversal of the self advantage when the self was assigned to the medium tone. In this case, then, there is a stronger ‘natural’ order along a conceptual self than a perceptual tone dimension.

These findings have several implications for the mental representation of the self within a social dimension and its comparison to representations of reward value. For rewards, there was a relative processing benefit when either endpoint of the physical (tone) and reward dimensions overlapped. This result is akin to those advantages in response speed found due to structural polarity correspondence (e.g., Proctor and Cho, 2006; Lakens, 2012) between physical and conceptual (or response) representations (when considering the medium points on the different dimensions as negative polar, as there were always greater costs compared to endpoints, and the two endpoints as positive polar). For the social association, however, there was only a benefit when the positive end of the perceptual dimension (self) overlapped with either endpoint of the physical dimension, while overlap between the negative endpoint (stranger) and endpoints of the physical dimension had no effect. Thus, the social dimension does not appear to be represented as a continuum with two marked endpoints (self and stranger) but rather as a dimension with a single endpoint (the self), or represented as two categories (self vs. other).

In general, the observed patterns between self, reward, and tone order were very similar in the absence of labels. Here we only assessed the effect of the label under multisensory conditions. However, it is just as likely that reward and self-biases will persist in the absence of explicit labels with unisensory visual or auditory stimuli. It is important to note that self-biases were significantly larger in the visual only than the multisensory labeled condition irrespective of tone order (i.e., unlike reward biases). Therefore, vision may dominate for self-associations; it may be easier to form self-associations with visual representations of objects than auditory and multisensory representations. Indeed, the absence of the label further reduced both self and reward biases in the multisensory condition. Future studies need to investigate whether the absence of labels similarly reduces self and reward biases with visual only self-associations.

Counterbalancing procedures are often used in psychophysical studies to control for order and other confounding effects (e.g., object shape or tone frequency). However, if the confounding effects are strong and biased, as in this case, then they may not balance out and can result in misleading interpretations. Pure tones have a natural physical hierarchy and have a strong influence on learnt conceptual hierarchies in higher cognition (in this case with self and rewards representations). Our study used a completely balanced design with equal participant numbers per group and condition. Therefore, we were still able to explore the unexpected effect of tone order on self and reward biases. Future studies will need to investigate whether visual object hierarchies (e.g., object size or value) also influence biases toward the self and reward.

## Conclusion

Both self and reward biases were observed with visual and multisensory stimuli even in the absence of labels following a brief learning phase. The results revealed marked differences in the underlying representational structure of social distance and rewards, and how they interact with perceptual representations that have a natural physical order. Furthermore, we would argue that outlining the representation of stimuli along a mental dimension may contribute to previous and future findings on self- and reward-advantages in perceptual matching.

## Data Availability Statement

The raw data supporting the conclusions of this article will be made available by the authors, without undue reservation.

## Ethics Statement

The studies involving human participants were reviewed and approved by the Department of Experimental Psychology, University of Oxford. The patients/participants provided their written informed consent to participate in this study.

## Author Contributions

MS and AB designed the experiments, collected and analyzed the data, and wrote the manuscript. CS made valuable comments and suggestions and assisted in the writing of the manuscript. All authors contributed to the article and approved the submitted version.

## Conflict of Interest

The authors declare that the research was conducted in the absence of any commercial or financial relationships that could be construed as a potential conflict of interest.
